# Factors associated with physical activity participation among children: a systematic review protocol

**DOI:** 10.1186/s13643-023-02226-0

**Published:** 2023-04-27

**Authors:** Prateek Srivastav, K. Vaishali, Eti Rajwar, Suzanne Broadbent, H. Vinod Bhat

**Affiliations:** 1 Department of Physiotherapy, Manipal College of Health Professions, Manipal Academy of Higher Education (MAHE), Manipal, Karnataka India; 2 Public Health Evidence South Asia, Prasanna School of Public Health, Manipal Academy of Higher Education (MAHE), Manipal, Karnataka India; 3 School of Health & Behavioural Sciences, University of the Sunshine Coast, Sippy Downs, QLD Australia; 4 The Apollo University, Chittoor, Andhra Pradesh India

**Keywords:** Physical activity, Exercise, Sports, Physical education, Children, Adolescents, Kids, Youth, Factors, Determinants

## Abstract

**Background:**

Reduced physical activity (PA) is one of the significant health concerns in adults and children alike. Despite the proven benefits of PA, most children, globally, do not meet the weekly criteria of enough PA to maintain health. The proposed systematic review is the review of the factors and will provide information on the factors associated with PA participation in children.

**Methods:**

The proposed systematic review will be conducted based on the methodology from the Cochrane Handbook for Systematic Reviews of Interventions. We will include observational studies (cross-sectional, case–control, and cohort studies), randomized controlled trials (RCTs), and non-randomized study designs for information on factors associated with PA participation among children. Studies with participants in the age range of 5–18 years, indulging in physical activity of 60 min per day for a minimum of 3 days a week, will be included. Studies including differently abled children, children under medical treatment, and those taking medications for illnesses such as neurological, cardiac, and mental health conditions will be excluded from the review. We will search MEDLINE (via PubMed and Web of Science), Scopus, EMBASE, CINAHL, Cochrane CENTRAL, and PEDro for English language publications published from the inception till October 2022. For additional studies, we will search websites such as the Australian Association for Adolescent Health International Association for Adolescent Health and a reference list of the included publications. Selection of studies, data extraction, and quality assessment of the included studies will be performed in duplicate. Quality assessment of the included studies will be performed using the Cochrane Risk of Bias tool (ROB-II) for RCTs, New-Castle Ottawa, for observational studies, and ROBINS-I (Risk of Bias for Non-Randomized studies of Interventions) for non-randomized study designs.

**Discussion:**

The proposed systematic review and meta-analysis will present a summary of the available evidence on factors associated with PA participation in children. The findings of this review will provide new insights into how exercise providers can improve PA participation among children and can also help healthcare workers, clinicians, researchers, and policymakers to plan long-term interventions targeting child health.

**Systematic review registration:**

PROSPERO CRD42021270057.

**Supplementary Information:**

The online version contains supplementary material available at 10.1186/s13643-023-02226-0.

## Background

Regular physical activity (PA) is essential for healthy adulthood and reducing the risk of chronic diseases such as hypertension, and metabolic and cardiovascular disorders [1, 2]. Regular PA in childhood helps to maintain normal body weight, improve muscular and cardiorespiratory health, and enhance brain development [3–9]. Thus, regular PA in childhood lowers the risk of chronic diseases in adulthood [2]. The American College of Sports Medicine (ACSM) recommends a minimum of 60 min of PA per day for a minimum of 3 days a week in children [10]. The recommendations suggest combinations of moderate and vigorous intensity activity such as walking, running, dancing, and cycling [10]. However, industrialization, modernization, and digitalization have contributed to a steep decrease in PA among children [11]. A systematic review conducted in 2018 by Sharara et al. reported that physical activity among Arab children and adolescents was significantly less than the prescribed ACSM guidelines [12]. Several recent reviews also reported the trends of decreased PA among children and adolescents worldwide [13, 14]. The World Health Organisation (WHO) published a global report on PA, and it concluded that 81% of adolescents aged 11–15 years do not meet the WHO recommendations of being physically active for health [15]. This physical inactivity has burdened public health indirectly as a physically inactive child is most likely to be an inactive adult [16]. A 2016 report by Martinez et al. showed that physical inactivity linked to increased morbidity and mortality costs the public health sector $53.8 billion worldwide [16].

The Institute of Medicine (IOM) proposed the use of the social-ecological model (SEM) for childhood obesity interventions [17]. This model categorizes factors influencing PA as an individual (e.g., age, gender, physical development), social (e.g., family, peers, education), environmental (e.g., green spaces, playgrounds, bicycle lanes), and policy-related (e.g., education, transport) [17]. Researchers hypothesize that such factors cumulatively affect the PA of an individual and should be included in interventions to bring about behavioral change and increase PA [17]. The influence of these factors on children’s PA has been individually investigated but not summarized systematically [18–20].

Reduced PA levels among children are a global health issue. Examining the magnitude of the influence of factors affecting PA participation among children will be valuable in setting priorities and designing interventions to increase PA in children and prevent chronic diseases in adulthood. Previous reviews have reported that children globally do not meet recommended levels of PA, but the specific contributing factors have not been identified. Our review will address the gap in the evidence by systematically reviewing published literature to establish the specific factors that influence PA participation in children. Summarizing the factors associated with PA participation in children can inform policymakers, researchers, and healthcare practitioners to formulate appropriate PA strategies and facilitation of PA adherence.

## Objectives

Primary objective

To identify the factors (enablers and barriers) associated with PA participation among children.

Secondary objective

To provide recommendations for increasing the PA participation among children (based on the findings of the primary objective)

## Methods

This systematic review protocol is based on the Preferred Reporting Items for Systematic Review and Meta-analysis Protocols (PRISMA-P) guideline [21], and the systematic review and meta-analysis will be conducted according to the Cochrane Handbook for Systematic Review of Interventions [22]. The protocol for the systematic review is registered in the International Prospective Register of Systematic Review (PROSPERO) with registration number CRD42021270057. Any amendments in the protocol will be documented in the final review.

### Eligibility criteria for study selection

#### Types of participants

The review will include studies assessing the influence of factors on children’s participation in PA, regardless of gender, ethnicity, and socio-economic status. The age groups for inclusion will be 5–18 years. Studies on differently abled children, children under medical treatment and those taking medications for illnesses such as neurological, cardiac and mental health conditions will be excluded from the review as the factors influencing differently abled and children under medical treatment will vary.

#### Exposure

The exposures in the review will be factors (both facilitators and barriers) influencing PA participation in children. The factors can be categorized as (a) individual factors known to influence one’s propensity for being physically active regularly [17]; (b) social environmental factors: the way one interacts socially and with the environment [17]; (c) policy factors: which include the regulatory laws, formal or informal, implied by government or organizations to support PA participation [17].

#### Dependent variable

The dependent variable is the participation of children in PA where PA is defined as any bodily movement increasing resting energy expenditure [10]. Studies with repeated bouts of physical activity or exercise to improve physical fitness, including multiple sessions over weeks, months, or years, will be eligible for inclusion. To be eligible for inclusion, PA should be a minimum of 60 min per day for a minimum of 3 days a week, primarily a combination of moderate-intensity to vigorous-intensity activities, causing a noticeable to a substantial increase in heart rate and breathing, respectively [10]. The PA should combine aerobic activities such as brisk walking, running, swimming, dancing, and strength training (e.g., tug of war, climbing a tree, or working with Thera bands) [10]. Studies reporting the transient effect of a single bout of exercise or PA will be excluded. Studies with PA interventions in school settings and extracurricular settings such as home, community, or other settings will be included.

#### Types of studies

Observational studies such as analytical cross-sectional studies, case–control studies, and longitudinal studies (e.g., cohort studies) will be included for information on factors associated with PA participation among children. We will also include experimental study designs such as randomized controlled trials (RCTs). In cases where RCTs are not possible due to the nature of the intervention or due to ethical concerns, we will include the non-randomized trials or the experimental studies not following randomization for the allocation of participants. Additionally, we will also include other non-randomized study designs such as controlled before and after studies (CBAs) and quasi-experimental studies.

#### Conceptual framework

**Fig. 1:**
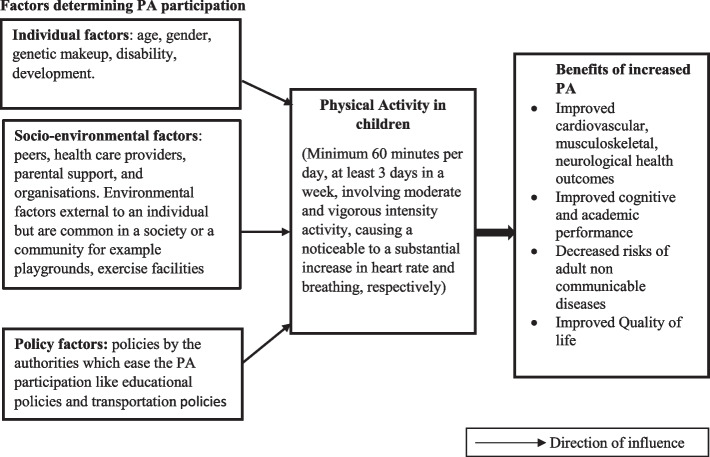
Conceptual Framework of direct and indirect factors effecting PA participation in children

We hypothesize several factors’ direct and indirect effects on PA participation in children through the conceptual framework (Fig. 1). Several factors (involving individual, social, and policy-related factors) directly or indirectly influence PA participation among children [23]. The individual factors are psychological, behavioral, and demographic factors and will include age, sex, likes or dislikes, education, socioeconomic status, self-efficacy, skill levels, and attitude towards PA [24]. As their age increases, children are less likely to be physically active [24]. Gender plays an essential role in participation because females tend to play less vigorous games than their male counterparts [25]. Educated children or parents tend to understand the importance of PA participation better than less educated or uneducated parents [26].

The social factors also play an essential role in PA participation [27]. The social factors can be interpersonal (peers, parents, teachers), organizational (schools, day-care, home, neighborhood), and community (worship place, geographical locality) [27]. A child is likely to be more active if the parents are physically active as compared to sedentary parents; also, a friend’s PA participation reinforces PA in individuals; therefore, peer validation of PA plays a key role, and children tend to be socially more interactive with physically active peers as compared to inactive or less active peers [28]. A habit of PA participation should be encouraged in children in schools, homes, and healthy neighborhoods [29]. Children who are part of dense, homogenous communities are more likely to be influenced by the norms and culture of that community [29].

Policy factors are another influence on PA participation behavior and are a crucial feature of public health [30]. Policies can range from the restrictive, such as banning exposure to sugar-based product advertisements to increasing health promotion resources such as parks and community exercise facilities [30]. These policies may increase PA participation among children [30]. We anticipate that modifying these factors will increase the PA participation in children and decrease obesity and sedentary lifestyle-related health risk factors.

### Searching for studies

We will search Medical Literature Analysis and Retrieval System Online (MEDLINE) via PubMed and Web of Science, Scopus, Excerpta Medica Database (EMBASE), Cumulated Index to Nursing and Allied Health Literature (CINAHL), The Cochrane Library (Cochrane Database of Systematic Reviews, Cochrane Central Register of Controlled Trials (CENTRAL), and Physiotherapy Evidence Database (PEDro) for English language publications, published from the inception of the database till November 2021. In addition, we will search the following websites: Australian Association for Adolescent Health (https://www.aaah.org.au/), International Association for Adolescent Health (https://iaah.org/, https://www.who.int/data/maternal-newborn-child-adolescent-ageing/documents/mca), Association of Adolescent and Childcare in India (https://www.aacci.in/), Society for Adolescent Health and Medicine (https://www.adolescenthealth.org/Home.aspx), The Childhood Obesity Foundation (https://childhoodobesityfoundation.ca/about/), and Centres for Disease Control and Prevention, (https://www.cdc.gov/obesity/data/childhood.html), for additional resources. We will search the reference list of the included publications for additional studies. Search keywords will be “Physical activity, exercise, sports, physical education, children, adolescents, kids, youth, factors and determinants.” We will use a combination of controlled vocabulary terms such as Medical Subject Headings (MeSH) and free text words for designing the search strategy. The initial search will be conducted in PubMed, and the search terms will then be modified according to the requirements for different databases. The preliminary search strategy for PubMed is given in Additional file 1*.*

### Selection of studies

Two reviewers (PS and ER) will independently screen the titles and abstracts of potential studies for eligibility based on the eligibility criteria of the review. Any disagreement at this stage will be resolved via discussion or in consultation with the third reviewer (SB). Full texts of all the included studies will be downloaded, and in case of non-available full texts study, authors or subject experts will be contacted for full texts: in case the full text remains unavailable, we will exclude that study. In case of two reports of the same study, the latest and the one with the complete information will be included for full-text and the other report will be marked as a duplicate. In the next step, a full-text screening will be performed by two review authors (PS and ER) independently. Any disagreement during this stage will be resolved by consensus or via contacting the third author (SB). More information about the process of selection of studies is given in Table 1.

**Table 1:** Screening protocol

**1) Title and abstract screening**		
A) Is the study published in English?	If “yes,” go to B	If “no,” exclude the study
B) Does the study involve one of the following designs or analyses: • Analytical cross-sectional studies, case–control studies • Longitudinal studies • Randomized controlled trials, and non-randomized trials	If the answer is “yes,” go to C OR if it is not clearly stated in the abstract, go to C	If the publication is a commentary, perspective, editorial, review, or conference abstract, exclude the study
C) Does the study explain PA as any physical activity/exercise for a minimum of 60 min in a day for a minimum of 3 days in a week. There is no restriction on the mode and setting of PA. It should include moderate or vigorous activity (causing a noticeable to a substantial increase in HR and breathing, respectively) Activities like such as brisk walking, running, swimming, dancing, and strength training (e.g., tug of war, climbing a tree, or working with Thera bands)	If the answer is “yes” OR if it is not clearly stated and you are in doubt, then include the study for a full-text screening and move to “D”	If “no,” exclude the study
**2) Full-text screening**		
D) Does the study involve children aged 5–18 years	If “yes,” go to E	If “no,” exclude the study OR if the study includes differently abled children, children under medical treatment, and those taking medications for illnesses such as neurological, cardiac, and mental health conditions
E) Does the study involve one of the following designs or analyses: analytical cross-sectional studies, case–control studies, longitudinal studies randomized controlled trials, and non-randomized trials	If the answer is “yes,” go to F	If no, exclude the study
F) Does the study explains the PA Any physical activity/exercise for a minimum of 60 min in a day for a minimum of 3 days in a week. There is no restriction on the mode and setting of PA. It should include moderate or vigorous activity (causing a noticeable to a substantial increase in HR and breathing, respectively) Activities like such as brisk walking, running, swimming, dancing, and strength training (e.g., tug of war, climbing a tree, or working with Thera bands)	If the answer is “yes,” then move to G OR if it is not clearly stated and in doubt: flag for discussion	If “no,” exclude the study
G) Does the study include outcomes of our interest? Such as (a) individual factors known to influence one’s propensity for being physically active regularly; (b) social environmental factors: the way one interacts socially and with the environment; (c) policy factors: which include the regulatory laws, formal or informal, implied by government or organizations to support PA participation. Secondary outcomes (a) categorization of enablers and barriers to physical activity according to WHO geographical regions; (b) strategies and interventions used to increase/promote physical activity among children	If the answer is “yes,” then include it for data analysis	If “no,” exclude the study

### Data management and extraction

Data will be managed using the EndNote reference manager and Microsoft Excel Office software. Data will be extracted by two review authors (PS and ER) independently by using the pre-tested data extraction sheet. All the reviewers will perform pilot testing of the data extraction sheet. Information related to bibliographic details, participants, PA, and outcome details will be extracted. An example of the data extraction form is given in Table 2. We plan to contact the study authors in case of any missing or unclear information. Any disagreements between the review authors will be resolved by discussion and input from a third reviewer (SB).

**Table 2:** Data extraction format

Characteristics	Details to be extracted
Publication details	Title
	First author’s last name
	Journal
	Year of publication
	Publication type: Funding source
Population characteristics	Age
	Gender
	Religion/race/ethnicity
	No of the participants included
Location	Country or other details of the place where the study was conducted
	Setting; community/home/school-based
Study methodology/design	Study design: analytical cross-sectional studies, case–control studies, longitudinal studies, RCTs, non-RCTs
	Aim of the study
	Method of data collection
	Recruitment and sampling methods
	Eligibility (inclusion and exclusion criteria)
	Type of analysis
Intervention details	Aerobic training
	Strength training
	Frequency
	Intensity
	Time
	Type
	Method of PA measurement (subjective or objective)
Outcome details	List down outcome, variable type: continuous or categorical, type of analysis, effect measures with 95% CI (such as OR, risk ratio, HR) No of participants analyzed, number list to follow-up
Limitations	
Others	

### Critical appraisal of the included studies risk of bias in individual studies

The reviewers will use the Cochrane Risk of Bias (ROB2) for assessing the quality of included RCTs [31]. Each selected publication will be checked for i) bias from the randomization process, (ii) bias due to deviations from intended interventions, (iii) bias due to missing outcome data, (iv) bias in the measurement of the outcome, and (v) bias in the selection of reported result. Each domain will be judged as “low risk,” “high risk,” or “risk with some concerns” with appropriate reasoning. The JBI Checklist for Analytical Cross-Sectional Studies’ is for assessing the quality of the analytical cross-sectional studies [32, 33]. The Newcastle Ottawa scale for assessing the quality of the observational studies such as case–control and cohort studies [34]. Quality assessment of non-randomized studies will be performed using the Risk Of Bias in Non-Randomized Studies-of Interventions (ROBINS-I) [35]. According to this tool, bias in a non-randomized trial will be assessed for the following seven domains: (a) bias due to confounding, (b) bias in the selection of participants into the study, (c) bias in the classification of interventions, (d) bias due to deviations from intended interventions, (e) bias due to missing data, (f) bias in the measurement of outcomes, and (g) bias in the selection of the reported result. The assessed risk will be categorized as low risk, moderate risk, serious risk, and critical risk. The risk of bias will be performed by two reviewers (PS and ER) independently, and any disagreement will be resolved by consensus or by consultation with the third reviewer (SB).

### Measure of effects

If continuous, data will be reported as mean and standard deviation with a 95% confidence interval (CI). For categorical data, estimates of odds ratio (OR) and risk ratio (RR) with 95% CI will be used for reporting.

Odds ratio will be extracted and reported for observational studies where the estimation of risk is difficult, such as analytical cross-sectional studies and case–control studies. Risk ratio (95%CI) will be extracted and reported for longitudinal studies (e.g., cohort studies) and experimental studies (RCTs and NRCTs). The final results will be reported as RR (95% CI), as relative risk or risk ratio is considered as a more intuitive measure of effect and is easy to interpret [22].

### Data synthesis

#### For primary objective

Findings of the review will be presented via “the characteristics of included studies” and “summary of findings” table (as shown in Table 1). Meta-analysis will be performed only if it is appropriate, i.e., for studies that are clinically and methodologically similar. Odds ratio and risk ratio will be computed as effect measures and will be pooled in a meta-analysis RR (95% CI) will be used to finally report the findings. In case all the studies provide only OR as an effect measure statistic, in this case, meta-analysis will be computed using OR and the final results will be re-expressed as RR [22]. Statistical heterogeneity will be determined using the chi-square (Q) statistic, where a *P* value < 0.1 will be considered as statistically significant. Statistical heterogeneity will be quantified using *I*-squared statistics, describing the percentage of the variability in effect estimates that is due to heterogeneity rather than chance [22]. Based on the *I* ^2^ statistic, the level of heterogeneity will be categorized as follows [22]:

1. 0 to 40%: might not be important
2. 30 to 60%: may represent moderate heterogeneity
3. 50 to 90%: may represent substantial heterogeneity
4. 75 to 100%: considerable heterogeneity

Random effects model with and inverse-variance approach, i.e., DerSimonian and Laird method will be used for meta-analysis [22]. This method given by DerSimonian and Lair in 1986 makes use of a “moment-based” estimate of the between-study variance [22]. The random effects model is chosen because of the assumed distribution of effects between the studies and as it is assumed that the results are not fixed [22]. The results of the meta-analysis will be reported via a forest plot.

A narrative synthesis will be performed in case of high heterogeneity and when meta-analysis is not possible, i.e., due to the presence of “clinical,” “methodological,” and “statistical.” In the narrative synthesis, the outcome and findings will be reported narratively, and tables/figures will be used for explanation.

If it is possible, sub-group analyses will be performed based on geographical location, age, gender, type of physical activity, etc. If sufficient data is available, we will perform sensitivity analysis for assessing the robustness of the results.

#### For secondary objective

The primary objective findings, i.e., the factors associated with PA participation among children will be used to frame the policy and practice recommendations. The different factors for PA participation will be listed, based on our contextual framework. Additionally, a list of recommendations present in the available scientific literature will be prepared. These findings will be further discussed with all the relevant stakeholders to frame the appropriate recommendations.

### Meta-biases

Funnel plots will be used for the identification of reporting or publication bias. The identification of reporting bias will be done based on a visual assessment of the funnel plot, where a symmetrical funnel plot will be interpreted as the absence of reporting bias and an asymmetrical plot will indicate the presence of reporting bias.

## Discussion

Physical inactivity is one of the leading risk factors for lifestyle diseases and increased morbidity worldwide. PA is a modifiable factor and increasing PA levels among children can help reduce health risk factors, promote healthy lifestyles, and improve their quality of life.

In this review, we expect to identify the factors associated with PA participation among children. We hope that systematically reviewing such factors will help address the paucity of evidence surrounding factors contributing to poor engagement with physical activity, which can lead to obesity and other medical conditions. Identifying the factors influencing PA participation in children and adolescents can provide guidance, health promotion, and resources for the families, policymakers, and authorities to improve the lifestyle and health of young people. Any major changes in the protocol will be amended in PROSPERO. An abstract of the complete synthesis will be presented in relevant conferences. The manuscript will be submitted to a relevant journal for publication. This systematic review will support the caregivers, policymakers, and healthcare providers by summarising the evidence for different behavioral interventions intended to increase the physical activity participation among children. This behavioral change is particularly relevant globally given WHO’s focus on preventing diseases in children.

## Supplementary Information

**Additional file 1. **PRISMA-P 2015 Checklist.

**Additional file 2. **Factors determining PA participation.

**Additional file 3.** Draft PubMed Search Strategy.

## Data Availability

Data sharing is not applicable to this protocol as datasets are not generated or analysed.

## Abbreviations

ACSM: American College of Sports Medicine
CBA: Controlled before-after studies
CI: Confidence interval
GDT: Guideline Development Tool
GRADE: Grading of Recommendations Assessment, Development and Evaluations
IOM: Institute of Medicine
MeSH: Medical Subject Headings
OR: Odds ratio
PA: Physical activity
PRISMA-P: Preferred Reporting Items for Systematic Review and Metanalysis Protocols
RCT: Randomized controlled trials
RoB2: Cochrane Risk-of-Bias tool
ROBINS-I: Risk Of Bias tool to assess Non-Randomized studies of Interventions
RR: Risk ratio
SEM: Social-ecological model
WHO: World Health Organization
