# Supplementary material for: Factors associated with physical activity participation among children: a systematic review protocol
Source: Syst Rev. 2023 Apr 27;12:70. doi: 10.1186/s13643-023-02226-0 (PMC10134558; doi:10.1186/s13643-023-02226-0)
Supplement: Supplementary file 2 — Additional file 2. Factors determining PA participation. [file 13643_2023_2226_MOESM2_ESM.pdf]

Additional file 2

Factors determining PA participation

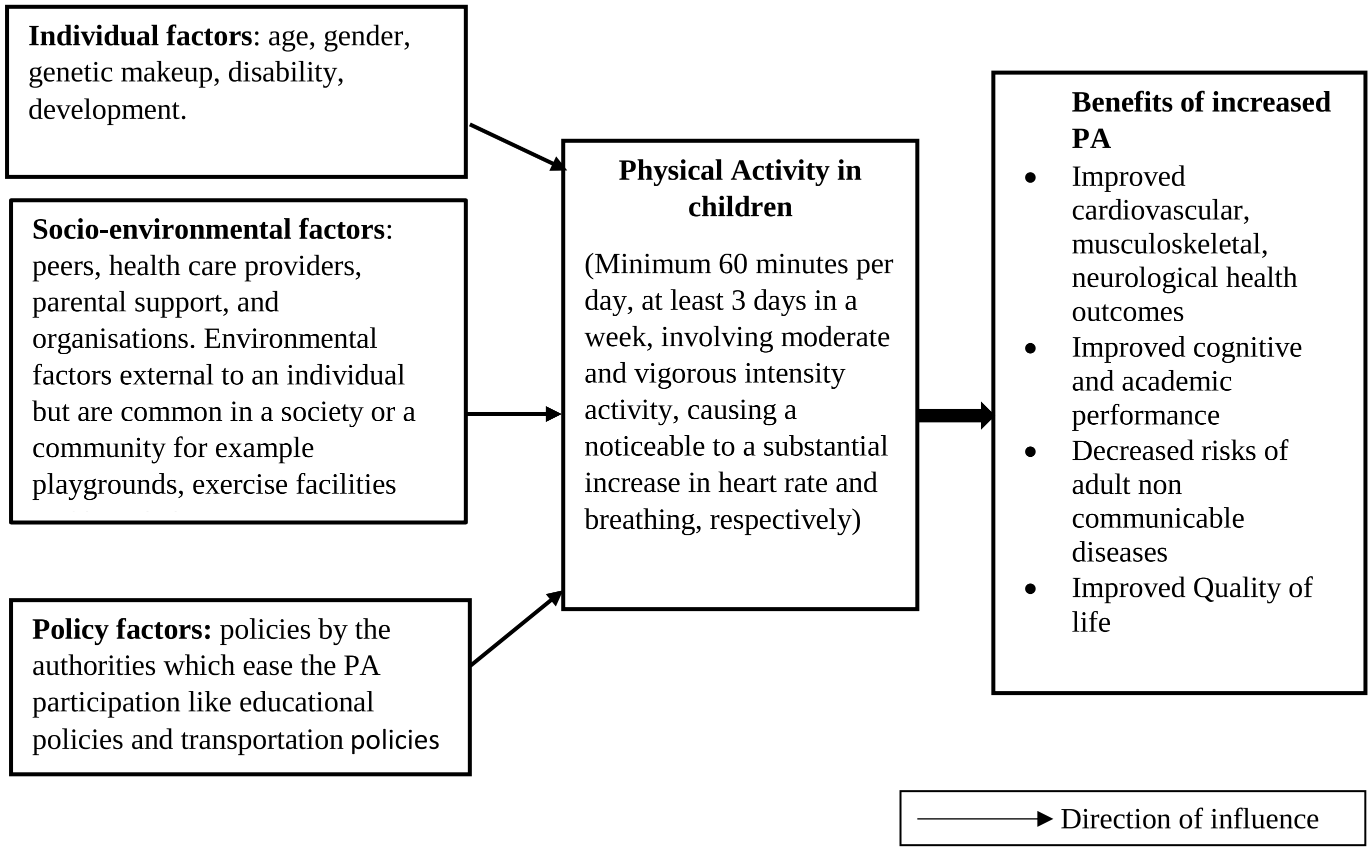

Figure 1: Conceptual framework for factors determining PA participation in children
