# Supplementary material for: Factors associated with physical activity participation among children: a systematic review protocol
Source: Syst Rev. 2023 Apr 27;12:70. doi: 10.1186/s13643-023-02226-0 (PMC10134558; doi:10.1186/s13643-023-02226-0)
Supplement: Supplementary file 3 — Additional file 3. Draft PubMed Search Strategy. [file 13643_2023_2226_MOESM3_ESM.docx]

**Additional File 3**

**Draft PubMed Search Strategy**

(("child"[MeSH Terms] OR "adolescent"[MeSH Terms] OR "child"[Title/Abstract] OR "children"[Title/Abstract] OR "adolescent*"[Title/Abstract] OR "teenager*"[Title/Abstract] OR "youth"[Title/Abstract]) AND ("exercise"[MeSH Terms] OR "sports"[MeSH Terms] OR "physical activity"[Title/Abstract] OR "physical activity participation"[Title/Abstract] OR "exercise"[Title/Abstract] OR "sport*"[Title/Abstract] OR "intense physical activity"[Title/Abstract] OR "vigorous physical activity"[Title/Abstract]) AND ("determinant*"[Title/Abstract] OR "barrier*"[Title/Abstract] OR "enabler*"[Title/Abstract] OR "facilitator*"[Title/Abstract] OR ("school-based physical education"[Title/Abstract] OR "family based health education*"[Title/Abstract] OR "college-based health education"[Title/Abstract] OR "physical activity promotion"[Title/Abstract]) OR "community-based health education"[Title/Abstract]) AND "english"[Language] AND ("humans"[MeSH Terms] AND "english"[Language]) AND ("humans"[MeSH Terms] AND "english"[Language])) AND ((humans[Filter]) AND (english[Filter]))
